# Binding affinities of human IgG1 and chimerized pig and rabbit derivatives to human, pig and rabbit Fc gamma receptor IIIA

**DOI:** 10.1371/journal.pone.0219999

**Published:** 2019-07-19

**Authors:** Maryam M. Bhatti, Allen G. Cai, Jan-Willem Theunissen

**Affiliations:** Iconic Therapeutics, South San Francisco, CA, United States of America; Icahn School of Medicine at Mount Sinai, UNITED STATES

## Abstract

While pigs and rabbits are used as models for human immune diseases, FcγR binding is poorly characterized in both test species. To evaluate antibody binding to FcγRIIIA, a receptor involved in antibody-dependent cellular cytotoxicity, chimerized antibodies were generated by grafting the variable regions of a human IgG1 onto scaffolds from both species. The affinities of the parent and chimeric antibodies to the FcγRIIIA proteins from all three species were determined. While the human IgG1 and rabbit IgG had similar affinities for each FcγRIIIA with notable differences across species, pig IgG1 only bound pig FcγRIIIA with appreciable affinity. Also, the functional pig and rabbit proteins described here can be used in future experiments, such as pharmacology and mechanism of action studies.

## Introduction

The pharmacology and mechanism of action of novel therapeutic biologics is evaluated in preclinical animal models as part of investigational new drug applications. For antibodies and recombinant proteins that contain the fragment crystallizable (Fc) region of an IgG, interpretation of the preclinical animal model studies can be confounded by immunogenicity and improper effector function [1].

As more antibodies and Fc-containing proteins are developed for chronic use, the impact of neutralizing antibodies in preclinical animal models can be partially overcome by the use of homologous proteins or surrogate antibodies [1, 2]. For antibodies, chimerizing the variable regions or grafting the complementary determining regions onto the framework of the test species can also be considered [1, 3].

In addition, antibodies and Fc-fusion proteins can rely on Fc effector function as a mechanism of action. For example, antibody-dependent cellular cytotoxicity (ADCC) of IgG1 antibodies has been reported in cancer and infectious diseases such as HIV and influenza [4–6]. The Fc region of IgG1 contains binding sites for the activating FcγRIIIA receptor expressed on NK cells and macrophages, immune cells that mediate ADCC [5]. When the binding of the Fc region to the test species’ FcγRIIIA is not sufficiently conserved, *in vivo* mechanism of action studies should be conducted with a surrogate or chimerized derivative.

Because pigs and rabbits are gaining traction as animal models of human infectious and other diseases [7–9], we established the affinities of a human IgG1 and chimerized derivatives to human, pig and rabbit FcγRIIIA by surface plasmon resonance (SPR). Human FcγRIIIA receptors, including the low affinity F158 and high affinity V158 variants, have been characterized extensively for binding to IgG’s [10, 11]. However, both pig and rabbit FcγR have only been isolated from immune cell preparations and minimally characterized [12, 13], making this the first study on recombinant expression, purification and characterization of pig and rabbit FcγRIIIA. With the advent of IgG Fc engineering in therapeutic proteins [14], ADCC-enhanced and -attenuated human IgG1 mutants were also included in the FcγRIIIA binding experiments.

## Results

### Generation and characterization of antibodies and FcγRIIIA proteins

Pig and rabbit chimeras of a human IgG1 antibody with a kappa light chain were created by grafting the variable regions of the heavy and light chains of a human anti–Tissue Factor antibody 25G1 [15] onto corresponding constant regions from pig and rabbit (S1 Table). While human and pig IgG1 is the predominant isotype in serum, rabbits only have one IgG isotype (one Cγ gene) [16, 17]. The kappa light chain is relevant in both test species, as the ratio of kappa versus lambda light chain usage is ~60:40 in human, ~50:50 in pig and ~95:5 in rabbit [18]. To evaluate the impact of Fc-engineering on the affinities for the different FcγRIIIA proteins in our SPR assays, ADCC-enhanced and -attenuated human IgG1’s (hIgG1-SD/IE and hIgG1-LA/LA/PG) were generated by using the previously described S239D/I332E and L234A/L235A/P329G mutations, respectively (S1 Table) [19, 20].

The FcγRIIIA receptors were expressed as extracellular domain (ECD) fragments (S2 Table). In humans the *FCGR3* gene family consists of *FCGR3A* and *FCGR3B*, with the latter containing a C to T change at 733, creating a stop codon and a 21 amino acid deletion at the C-terminus of FcγRIIIB [21]. While the rabbit FcγRIII sequence identified in our alignment search is annotated as FcγRIIIB, the protein sequence does not contain a C-terminal deletion. Therefore, this rabbit protein sequence is likely the homologue of human FcγRIIIA, and we referred to this sequence as rabbit FcγRIIIA henceforth.

**Fig 1:**
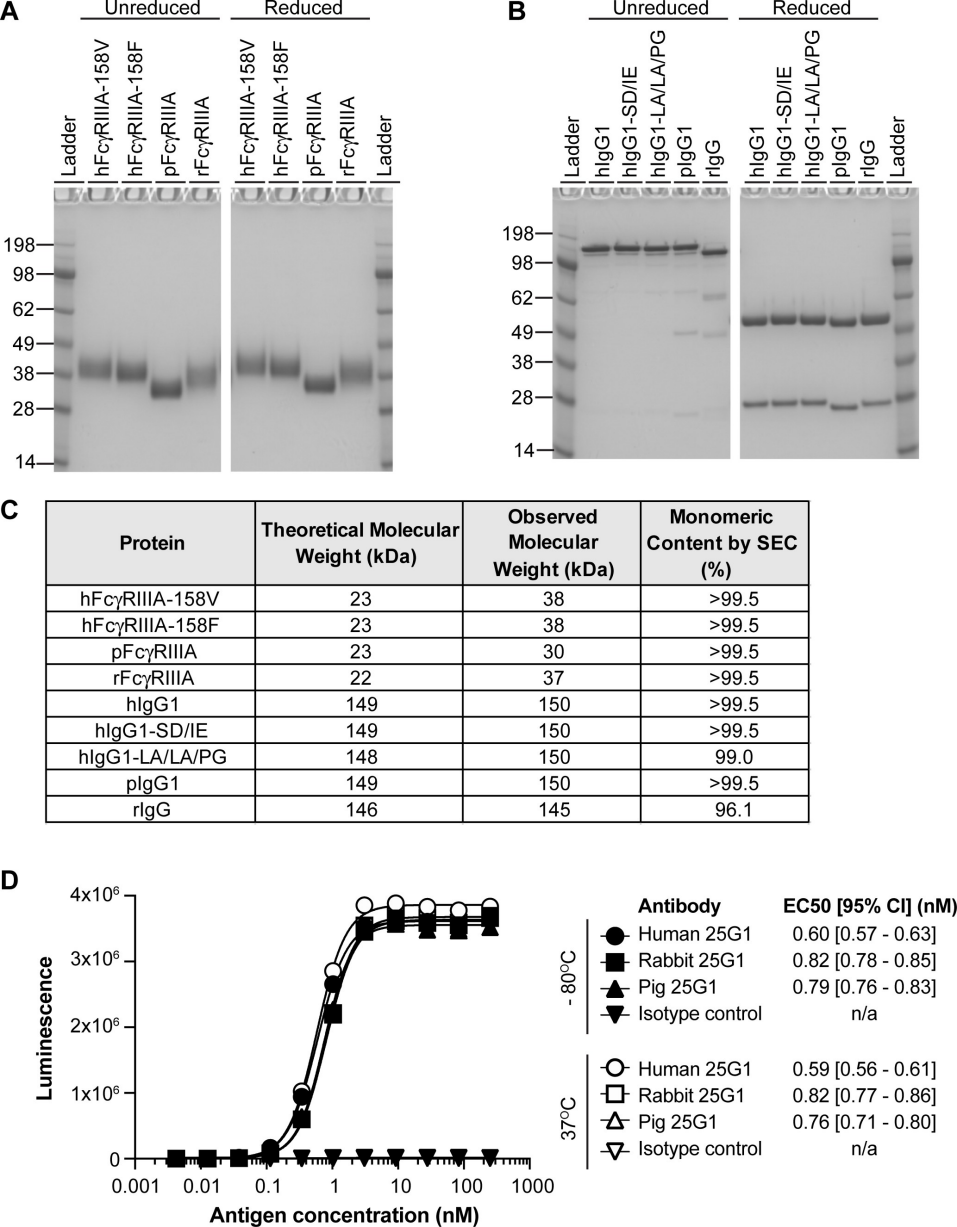
Expression and characterization of purified FcγRIIIA and antibodies. **A,** Coomassie stain of hFcγRIIIA-V158, hFcγRIIIA-F158, pFcγRIIIA, and rFcγRIIIA loaded at 2.5 μg per lane under non-reducing and reducing conditions. Molecular weight of ladder identified in kilodaltons (kDa) on left of gel. **B,** Coomassie stain of hIgG1, hIgG1-SD/IE, hIgG1-LA/LA/PG, pIgG1, and rIgG loaded at 2.5 μg per lane under non-reducing and reducing conditions. Molecular weight of ladder identified in kilodaltons (kDa) on left of gel. **C,** Molecular weight based on amino acid sequence, observed molecular weight based on Coomassie stain, and monomeric content based on analytical size exclusion chromatography (SEC). **D,** Binding of a titration of the Tissue Factor antigen to immobilized chimeric and parent antibodies. Reportable EC50’s and their 95% confidence intervals are listed. The antigen did not bind the isotype control antibody. Antibody preparations stored at—80°C or 37°C for 3 days were used in the assay. Filled symbols, antibodies stored at -80°C; open symbols, antibodies stored at 37°C. Circles: human 25G1; squares: rabbit 25G1; triangles: pig 25G1; upside-down triangles: isotype control.

The purified antibodies and FcγRIIIA proteins were characterized by gel electrophoresis and analytical size exclusion chromatography (SEC) (Fig 1). While the theoretical molecular weights of the FcγRIIIA ECD fragments were equivalent across species, the observed molecular weights of the human and rabbit FcγRIIIA proteins were slightly greater than the pig FcγRIIIA protein (Fig 1A and 1C). Monomer content by SEC for all proteins was greater than 95% (Fig 1C).

To ensure antigen-binding was not compromised by chimerization, chimeric pig and rabbit antibodies were tested alongside the parent human IgG1 for binding to human Tissue Factor (Fig 1D). The EC50’s of the chimeric antibodies were within two-fold of the human antibody, indicating that chimerization did not substantially impact antigen binding. In addition, stability of the parent and chimeric antibodies was assessed by comparing the antigen-binding of preparations stored at -80 or at 37°C for 72 hours (Fig 1D). An elevation in temperature did not affect the antibodies’ EC50s by more than 15%.

### Human, pig and rabbit IgG affinities for FcγRIIIA

To enable binding between the Fc region of the IgG antibodies and the FcγRIIIA ECD fragments, the kappa light chains were captured onto a carboxymethylated dextran chip immobilized with Protein L, a surface protein from *Peptostreptococcus magnus* known to bind antibodies through the kappa light chain [22]. The binding stability of the chip was first tested by capturing a five-point titration of hIgG1 in duplicate on flow cell 2 (FC2), demonstrating the ability of hIgG1 to consistently bind the surface after repeated regenerations (S1 Fig). To ensure antibodies were able to bind the sensor chip for use in a capture experiment, a three-point titration of all five antibodies was captured on FC2 and allowed to dissociate from the surface for 600 s (S1 Fig). The capture levels were similar for all antibodies, indicating that Protein L effectively interacted with the fully human and chimerized kappa light chains (S1 Fig). In addition, very little to no change in response units was observed throughout the dissociation step for all five antibodies, thereby precluding a confounding effect of capture dissociation during kinetic experiments (S1 Fig). Non-specific binding of FcγRIIIA to the protein L surface was not observed with a three-point titration of hFcγRIIIA-V158, with a response of less than 1 resonance unit (RU) (S1 Fig).

**Fig 2:**
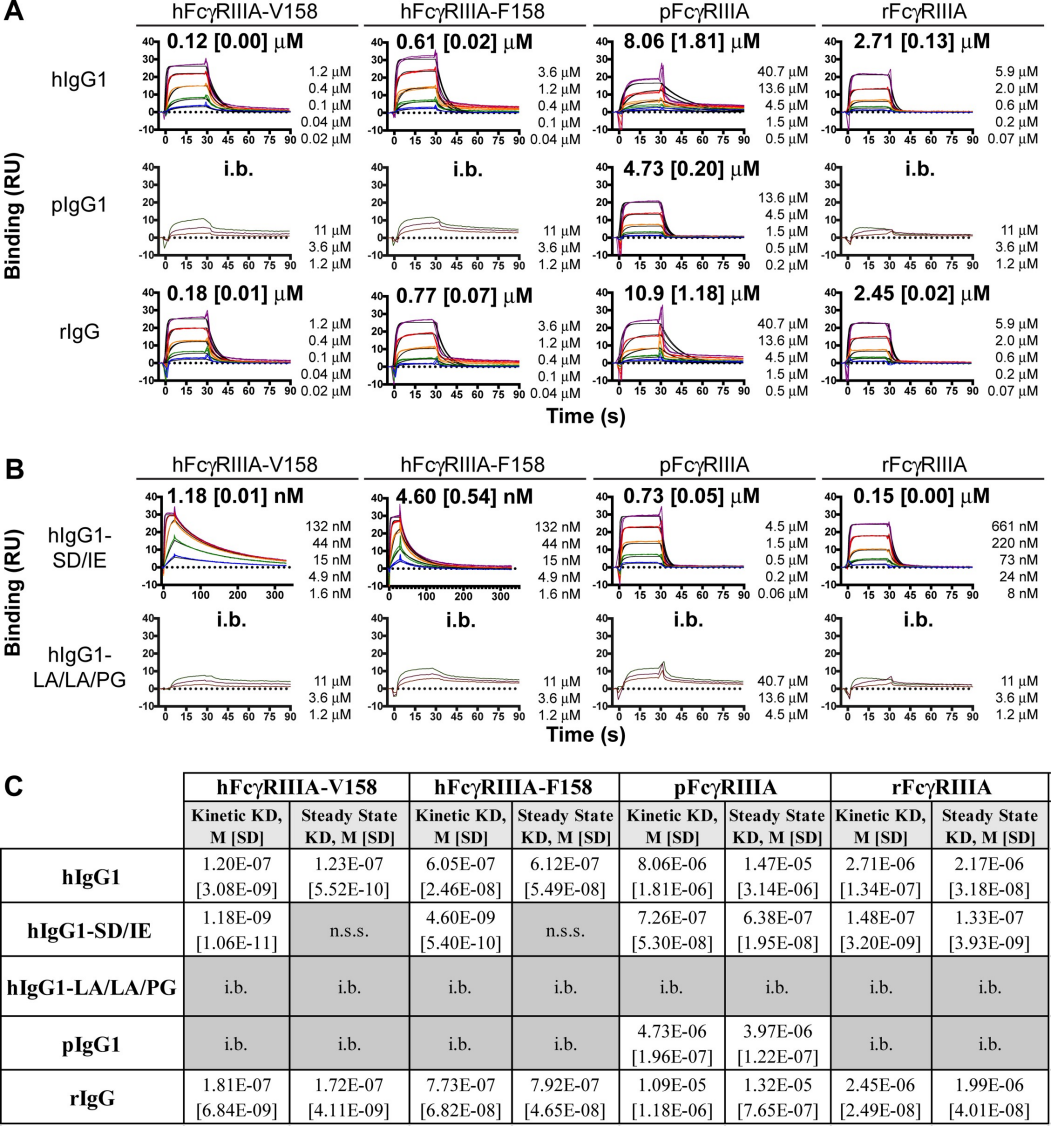
Binding of FcγRIIIA to human and chimeric antibodies. **A** and **B,** Sensorgrams of FcγRIIIA binding to hIgG1, pIgG1, and rIgG (**A**) and ADCC-enhanced and -attenuated hIgG1 (**B**). Kinetic fits are shown in black and titration concentrations are in color. The FcγRIIIA concentrations included in each titration are listed to the right-hand side of each sensorgram. Kinetic-based affinity constants determined using 1:1 Langmuir binding model are displayed above each sensorgram with standard deviation of three replicates in square brackets. **C,** Table of affinity values determined using either a kinetic model (1:1 Langmuir binding) or a steady-state affinity model with standard deviation of three replicates in square brackets. n.s.s = no steady state; i.b. = insufficient binding, affinity too weak for exact determination.

Initial kinetic experiments for all possible IgG–FcγRIIIA pairs were conducted to determine a suitable analyte concentration range based on the ability to recover at least seventy-percent of the theoretical analyte binding capacity (R_max_). For a subset of the IgG–FcγRIIIA pairs, less than 50% of the theoretical R_max_ was recovered, even at high analyte concentrations, limiting the ability to determine kinetic parameters by this method (referred to as IgG–FcγRIIIA pairs with insufficient binding (i.b.) in Fig 2A & 2B, S3 Table). For the rest of the IgG–FcγRIIIA pairs, kinetic parameters were determined from a five-point titration measured in triplicate and analyzed using a 1:1 Langmuir Binding Model (Fig 2A & 2B, S3 Table).

Accuracy of the kinetic K_D_ values was corroborated by steady-state K_D_ analysis [23]. For the IgG–FcγRIIIA pairs that reached steady-state for more than 10 s across all five analyte concentrations, steady-state affinities were derived (Fig 2C, S4 Table). The K_D_ values obtained from this steady-state evaluation were within two-fold of the K_D_ values obtained from the kinetic evaluation of the association and dissociation rates (Fig 2C, S3 & S4 Table). Importantly, the good agreement between the kinetic and steady-state K_D_ was maintained for the IgG–FcγRIIIA pairs for which the kinetic dissociation constant limit of the Biacore X100 was reached—k_d_ of 0.1 s^-1^ (S3 Table). In addition, the kinetic and steady-state K_D_ agreement was also maintained for the two IgG–FcγRIIIA pairs (hIgG1–pFcγRIIIA and rIgG–pFcγRIIIA) for which the fit of the 1:1 Langmuir Binding Model was suboptimal (i.e., a Chi^2^>5 compared to <3 for the other interactions) (S3 Table).

To ensure FcγRIIIA binding was not compromised by chimerization, biotinylated rabbit IgG was immobilized onto a streptavidin senor chip and binding of rabbit FcγRIIIA was assessed. Using this immobilization technique, steady-state affinities of 1.32 μM were obtained, compared to 2.45 μM with the chimeric antibody (S2 Fig). Therefore, the chimerization technique used herein does not appear to impact the intra-species interaction between antibody and FcγRIIIA.

Overall, when inspecting the IgG–FcγRIIIA interaction matrix in Fig 2A & 2B, a number of interactions stand out. First of all, each intra-species IgG–FcγRIIIA pair was able to bind with single-digit to sub micromolar affinity (Fig 2A). Thus, the intra-species IgG–FcγRIIIA interaction is conserved, albeit with different affinities. Secondly, the human IgG1 and rabbit IgG had similar kinetic K_D_ values for each FcγRIIIA, with a 40% difference for hFcγRIIIA-V158, a 24% difference for hFcγRIIIA-F158, a 30% difference for pFcγRIIIA, and a 10% difference for rFcγRIIIA (Fig 2A, S3 Table). Thirdly, the pig IgG1 bound pFcγRIIIA with a kinetic K_D_ of 4.73 μM, with no appreciable binding to human and rabbit FcγRIIIA (Fig 2A, S3 Table). Finally, the ADCC-attenuated and -enhanced hIgG1’s exhibited expected binding properties for the two human FcγRIIIA variants. The LA/LA/PG mutations, which abrogate ADCC activity [20], abolished binding to FcγRIIIA from all three species. On the other hand, the SD/IE mutations, which increase ADCC activity [19], improved the K_D_ of human IgG1 by at least one hundred–fold for the two human FcγRIIIA variants, and by at least ten-fold for pig and rabbit FcγRIIIA (Fig 2B, S3 Table). While the SD/IE mutations in hIgG1 improved both the k_a_ and k_d_ for the two human FcγRIIIA variants, SD/IE improved only the k_a_ for pig and rabbit FcγRIIIA, with no substantial impact on the k_d_ for rFcγRIIIA and a seven-fold deterioration of the k_d_ for pFcγRIIIA (Fig 2B, S3 Table). Overall, this data set highlights the need to interrogate each possible intra- and interspecies IgG–FcγRIIIA pair, not only for affinity, but also for kinetic parameters.

## Discussion

Here, the kinetics of human IgG1, pig IgG1 and rabbit IgG for human, pig and rabbit FcγRIIIA were derived. The affinities of the hIgG1–hFcγRIIIA pairs were largely in agreement with the literature, with some variation possibly due to differences in recombinant protein source, and/or SPR methodology and instrumentation [10, 11, 19, 23]. Whereas affinities of 120 and 1.2 nM were derived for the hIgG1–hFcγRIIIA-V158 and hIgG1-SD/IE–hFcγRIIIA-V158 pairs in this study, affinities of 252 and 2 nM were reported for these two pairs using trastuzumab as the hIgG1 [19]. Importantly, the approximately 100-fold difference between the hIgG1–hFcγRIIIA-V158 and hIgG1-SD/IE–hFcγRIIIA-V158 affinities was reproducible. Furthermore, the ~5-fold difference in the affinities of hIgG1–hFcγRIIIA-V158 (0.12 μM) and hIgG1–hFcγRIIIA-F158 (0.61 μM) was also previously reported [10, 11].

Intra-species binding characteristics of the pig and rabbit IgG–FcγRIIIA pairs were established for the first time. While the hIgG1–hFcγRIIIA affinity was 0.61 μM, the pIgG1-pFcγRIIIA and rIgG–rFcγRIIIA affinities were weaker, at 4.73 and 2.45 μM, respectively. Binding of the Fc of IgG1 to FcγRIIIA is mediated not only by protein-protein interactions, but also by carbohydrate-protein and carbohydrate-carbohydrate interactions [24, 25]. In this study, the antibodies and FcγRIIIA ECD fragments were produced in a human kidney–derived cell line. Because species and cell lineage affect glycosylation and affinity [26, 27], future studies should investigate differences in glycosylation between human, pig and rabbit.

Aside from putative differences in glycosylation machinery, glycosylation sites are not conserved between the three species. Whereas the N297 Fc glycosylation site is conserved across all three species, the FcγRIIIA glycosylation sites are not (S3 & S4 Figs). Out of the five reported N-linked glycosylation sites (N38, N45, N74, N162 and N169) in hFcγRIIIA [24], three and only one of these sites are conserved in rFcγRIIIA (N38, N45, N162) and pFcγRIIIA (N45), respectively (S4 Fig). In particular, an N-glycan at N162 of hFcγRIIIA extensively contacts one of the two Fc glycans [24, 25]; N162 is conserved in rFcγRIIIA, but not in pFcγRIIIA (S4 Fig). This lack of glycosylation site conservation correlated with different observed molecular weights of the FcγRIIIA proteins (Fig 1).

Inter-species cross-reactivity of the antibodies and hFcγRIIIA fragments was conducted to understand whether chimerization of lead hIgG1 therapeutic antibodies is needed for *in vivo* ADCC assessments in pig and rabbit. In a crystal structure of a human hFcγRIII fragment in complex with a Fc fragment of human IgG1 (hFc1), hFcγRIII bound hFc1 at the center of its horseshoe opening, making asymmetric contacts to the lower hinge regions of both the A and B chains of the hFc1 [28, 29]. About half of the amino acids in the hFcγRIII that interface with hFc1 are not identical in rabbit and pig FcγRIIIA (S4 Fig). However, the two tryptophan residues in the tryptophan-proline sandwich formed by W87 and W110 of FcγRIIIA and P329 of hFc1-B are conserved across all three species (S3 & S4 Figs). Overall, these amino acid changes in FcγRIIIA correlated with 13-fold and 4-fold reduced affinities of hIgG1 for pig and rabbit FcγRIIIA relative to hFcγRIIIA, indicating that chimerization is likely needed to enable evaluation of *in vivo* ADCC activity. Indeed, a recent study showed lack of *in vivo* ADCC activity of a hIgG1 in pigs [30].

On the Fc side of the hFcγRIIIA–hFc1 crystal structure, residues L234-S239 in the lower hinge were reported to dominate interactions with hFcγRIII [28, 29]. Complete conservation of the lower hinge between hIgG1 and rIgG correlated with similar affinities of hIgG1 and rIgG for the two human FcγRIIIA variants (Fig 2A). On the other hand, amino acids L234, L235 and G236 were not conserved in pIgG1 (S3 Fig), correlating with a lack of measurable affinities between pIgG1 and the two hFcγRIIIA variants (Fig 2A).

In humans and pigs, the IgG1 isotype is the most abundant serum IgG [16, 31]. Therefore, this study focused on chimerizing human variable regions onto the pig IgG1 isotype. With six isotypes present in pig, future studies should investigate the affinity between the other pig isotypes and FcγRIIIA [31].

The protein L capture method enabled IgG–FcγRIIIA affinity measurements through reproducible capture of antibody light chains and recovery of expected analyte binding capacity. Previous studies utilized anti-His capture of His-tagged FcγR, amine coupling of FcγR, and anti-Fab capture of antibodies to study IgG–FcγR kinetics [23, 24]. Because of its specificity to kappa light chains, protein L capture provides an alternate capture format for IgG–FcγR binding experiments. For example, to study C1q binding to Fc, the protein L capture method demonstrated a lack of Fc binding interference in comparison to protein A and protein G [32].

In summary, this study presented affinities for functional pIgG1–pFcγRIIIA and rIgG–rFcγRIIIA protein pairs. These pig and rabbit IgG and FcγRIIIA proteins can also be used for future immunological studies, including intra- and interspecies crystal structures that could shed more light on the conservation of IgG–FcγRIIIA interactions.

## Materials and methods

### Antibodies and proteins

The human IgG1 and kappa sequences were derived from the pFUSE-CHIg-hG1 and pFUSE2-CLIg-hk expression vectors, respectively (Invivogen). The pig IgG1 heavy chain and kappa light chain and rabbit IgG heavy chain and kappa light chain sequences were synthesized based on entries from IMGT, the international ImMunoGeneTics information system [33] (S1 Table). An alignment search with BLASTP 2.8.1 [34] using the human IgG1 constant region sequence as query against Reference proteins (refseq_protein) from *Sus scrofa* resulted in the identification of a protein sequence (NP_998993.1) that matched IMGT accession number AB699686, the *Sus scrofa* genes for immunoglobulin heavy chain variable and constant regions of clone L264P10, an antibody of the IgG1 isotype subclass [35]. *Oryctolagus cuniculus* only expresses a single IgG isotype (referred to as IgG), but contains two kappa subtypes (K1 and K2). The K2 subtype was used in this study, as the K1 subtype contains an additional disulfide bridge that links VL and CL [17]. Briefly, the pig heavy chain constant region was cloned into pFUSE-CHIg-hG1 containing the 25G1 variable region using the 5’ AfeI cloning site at the junction between the variable region and the constant region and the 3’ HpaI cloning site downstream of the coding region; the pig light chain (both the variable and constant region) was fully synthesized and cloned into pFUSE2-CLIg-hk using the existing 5’ AgeI and 3’ HpaI cloning sites; the rabbit heavy chain constant region was cloned into pFUSE-CHIg-hG1 containing the 25G1 variable region using the 5’ KpnI cloning site at the junction between the variable region and the constant region and the 3’ HpaI cloning site downstream of the coding region; the rabbit light chain (both the variable and constant region) was fully synthesized and cloned into pFUSE2-CLIg-hk using the existing 5’ AgeI and 3’HpaI cloning sites. Expression and purification of the antibodies was accomplished using described methodology [36]. After purification on a MabSelect Protein A column (GE Healthcare Bio-Sciences, 11-0034-94), all antibodies were buffer exchanged into phosphate-buffered saline (PBS) pH 7.4.

The human and pig FcγRIIIA sequences were obtained from the Uniprot Consortium [37]: human (P08637 for F158 variant and P08637 dbSNP:rs396991 for V158 variant) and pig (Q28942) [31]. The rabbit FcγRIIIA sequence (XP_002715293.1) was acquired by conducting a BLASTP alignment search with human FcγRIIIA (P08637) as query against Reference proteins from *Oryctolagus cuniculus*. The extracellular domain (ECD) fragments of the FcγRIIIA receptors were synthesized and cloned into pcDNA3.1V5-HisA (ThermoFisher Scientific, V81020) with a C-terminal 6x Histidine tag (S2 Table). Expression and purification of the FcγRIIIA ECD fragments was accomplished using described methodology [15]. Proteins were buffer exchanged into PBS pH 7.4, and monomeric human and pig FcγRIIIA ECD fragments were isolated by preparative size exclusion chromatography (GE Healthcare Bio-Sciences, 28-9909-44).

### Coomassie staining

Proteins were analyzed by electrophoretically separating 2.5 μg of unreduced or reduced protein on a 4–12% Bis-Tris gel with Bolt MES SDS buffer (ThermoFisher Scientific, B0002) and Coomassie staining the gel with the Pierce Power Stainer (ThermoFisher Scientific, 22833). The stained gel was imaged on an Amersham Imager 600 (GE Healthcare Bio-Sciences).

### Analytical size exclusion chromatography

Antibodies and proteins were characterized on a Vanquish UHPLC system (ThermoFisher Scientific) with an Acquity UPLC SEC, 200 Å, 1.7 μm particle size, 4.6 mm x 150 mm column (Waters Corporation, 186005225) as stationary phase, and 100 mM Sodium Phosphate in 150 mM Sodium Chloride pH 6.8 as mobile phase. Resulting chromatograms were analyzed using the Chromeleon 7.2 software’s Cobra Peak Detection algorithm (ThermoFisher Scientific). Based on retention times of an antibody reference standard and a gel filtration standard spanning 1.35 kDa to 670 kDa (Bio-Rad, 1511901), peaks were identified as either aggregate, monomer, or fragment.

### Enzyme-linked immunosorbent assay (ELISA)

Antigen-binding of the human and chimeric antibodies was assessed by ELISA using antibody preparations stored at -80°C or 37°C for 72 hours. High-binding 384-well plates (Greiner #781097) were coated overnight with a 67 nM of antibody solution at 20 μl per well, followed by one hour of blocking in PBS with 0.5% bovine serum albumin. After decanting the blocking buffer, an 11-point 1:3 titration of human Tissue Factor with a C-terminal 6x Histidine tag was added to the plates for 1 hour. Plates were then washed with 0.05% Tween20 in PBS and incubated for 30 minutes with a secondary anti-Histidine conjugated to horseradish peroxidase (Miltenyi # 130-092-783). After washing again, a chemiluminescent substrate was added (Thermo Fisher Scientific #37069) and luminescence was read on a Perkin Elmer Envision Plate Reader. Resulting data was graphed in Prism 8.0.0 (GraphPad) and EC50’s were estimated using a 4-parameter log fit.

### Surface plasmon resonance (SPR) assays

Kinetic measurements were collected on a Biacore X100 (GE Healthcare Bio-Sciences) at 25°C using HBS-EP+ Running Buffer (0.01 M HEPES pH 7.4, 0.15 M NaCl, 3 mM EDTA, 0.05% v/v Surfactant P20) and a Protein L Sensor Chip (GE Healthcare Bio-Sciences, 29205137). Antibody was diluted to 5.3 nM and captured on flow cell (FC) 2 for 24 seconds at 30 μl/min. FcγRIIIA was then injected for 30 s at 30 μl/min over both FC 1 and FC 2 and allowed to dissociate from the surface for either 60 s or 300 s. The surface was regenerated with a 5 μl/min, 120 s injection of 10 mM Glycine-HCl pH 1.7 between each cycle. Data was double referenced by subtracting FC1 from FC2 for all samples and then subtracting the average of two buffer sensorgrams collected at the start and end of the titration from analyte sensorgrams. Large spikes incurred from subtraction, between 1 to 3 seconds before and after the injection, were removed prior to sensorgram analysis.

Analyte sensorgrams were globally fit with a 1:1 Langmuir Binding Model using the Biacore X100 Evaluation software. Because samples were sufficiently diluted in running buffer and a reference surface was used, bulk refractive index contributions were set to zero. Kinetic fits and associated sensorgrams were exported from the Biacore Evaluation software and graphed in Prism 8.0.0 (GraphPad). A subset of sensorgrams were fit using a steady-state affinity model in the Biacore X100 Evaluation software, also setting bulk refractive index contributions to zero. Sensorgrams were considered appropriate for steady-state affinity analysis if an average change of less than 1 RU was observed over the last ten seconds of association.

To select an appropriate concentration range for a five-point titration, ligand-analyte pairs were first screened with three-point titrations of FcγRIIIA to determine a concentration at which at least 70% of the theoretical analyte binding capacity (R_max_) could be achieved experimentally. After each chosen five-point titration was assayed three times, each replicate titration was fit individually, and the resulting average and standard deviation were reported.

Due to the weak interaction between the fully rabbit kappa light chain and Protein L, a streptavidin-biotin format was used to measure affinity between fully rabbit IgG and rabbit FcγRIIIA. Biotinylated rabbit IgG (Jackson 011-060-003) was immobilized on FC2 of a Sensor Chip SA (GE Healthcare Bio-Sciences BR100398) and affinity measurements were performed using a titration of rabbit FcγRIIIA injected for 30 s at 30 μl/min over both FC 1 and FC 2 with a 60 s dissociation. Because the response returned to within 2% of baseline after each dissociation, the surface was not regenerated between injections. Data was double referenced by subtracting FC1 from FC2 for all samples and then subtracting the average of two buffer sensorgrams collected at the start and end of the titration from analyte sensorgrams. Steady-state affinity analysis was performed on each titration and the average of a triplicate is reported.

## Supporting information

S1 Table Antibody constant region sequences. (PDF)

S2 Table FcγRIIIA sequences. (PDF)

S3 Table Kinetic parameters derived from 1:1 Langmuir binding model. (PDF)

S4 Table Affinity values derived from steady-state binding analysis. (PDF)

S1 Fig Characterization of Protein L sensor chip. (PDF)

S2 Fig Steady-state affinity of rabbit IgG to rabbit FcγRIIIA using SA sensor chip. (PDF)

S3 Fig Clustal omega alignment of hIgG1, rIgG, and pIgG1 heavy chain (A) and light chain (B) constant regions with constant region numbering based on the human heavy and light chains.
(PDF)

S4 Fig Clustal omega alignment of hFcγRIIIA-V158, pFcγRIIIA, and rFcγRIIIA ECD fragments. (PDF)
