# Supplementary material for: Binding affinities of human IgG1 and chimerized pig and rabbit derivatives to human, pig and rabbit Fc gamma receptor IIIA
Source: PLoS One. 2019 Jul 19;14(7):e0219999. doi: 10.1371/journal.pone.0219999 (PMC6641210; doi:10.1371/journal.pone.0219999)
Supplement: S1 Table — (PDF) [file pone.0219999.s001.pdf]

S1 Table: Antibody constant region sequences

Protein	IgG1 constant region	Fc effector function	Sequence	Sequence reference (Source)
Human IgG1	IgG1 heavy chain	Wild-type	ASTKGPSVFPLAPSSKSTSGGTAALGCLVKDYFPEPVTVSWNSGALTSGVHTFPAVLQSSGL YSLSSVVTVPSSSLGTQTYICNVNHKPSNTKVDKKVEPKSCDKTHTCPPCPAPELLGGPSVFL FPPKPKDTLMISRTPEVTCVVVDVSHEDPEVKFNWYVDGVEVHNAKTKPREEQYNSTYRVV SVLTVLHQDWLNGKEYKCKVSNKALPAPIEKTISKAKGQPREPQVYTLPPSREEMTKNQVSL TCLVKGFYPSDIAVEWESNGQPENNYKTTPPVLDSDGSFFLYSKLTVDKSRWQQGNVFSCS VMHEALHNHYTQKSLSLSPGK	pfuse-hchg1 (Invivogen)
	Kappa light chain	N/A	RTVAAPSVFIFPPSDEQLKSGTASVVCLLNNFYPREAKVQWKVDNALQSGNSQESVTEQDSK DSTYSLSSTLTLSKADYEKHKVYACEVTHQGLSSPVTKSFNRGEC	pfuse2-hclk (Invivogen)
Human IgG1-SD/IE	IgG1 heavy chain	ADCC-enhanced, S239D/I332E (SD/IE)	ASTKGPSVFPLAPSSKSTSGGTAALGCLVKDYFPEPVTVSWNSGALTSGVHTFPAVLQSSGL YSLSSVVTVPSSSLGTQTYICNVNHKPSNTKVDKKVEPKSCDKTHTCPPCPAPELLGGP**D**VFL FPPKPKDTLMISRTPEVTCVVVDVSHEDPEVKFNWYVDGVEVHNAKTKPREEQYNSTYRVV SVLTVLHQDWLNGKEYKCKVSNKALPAP**E**EKTISKAKGQPREPQVYTLPPSREEMTKNQVSL TCLVKGFYPSDIAVEWESNGQPENNYKTTPPVLDSDGSFFLYSKLTVDKSRWQQGNVFSCS VMHEALHNHYTQKSLSLSPGK	Lazar et al., 2006 (PNAS)
	Kappa light chain	N/A	RTVAAPSVFIFPPSDEQLKSGTASVVCLLNNFYPREAKVQWKVDNALQSGNSQESVTEQDSK DSTYSLSSTLTLSKADYEKHKVYACEVTHQGLSSPVTKSFNRGEC	pfuse2-hclk (Invivogen)
Human IgG1-LA/LA/PG	IgG1 heavy chain	ADCC-attenuated, L234A/ L235A/P329G (LA/LA/PG)	ASTKGPSVFPLAPSSKSTSGGTAALGCLVKDYFPEPVTVSWNSGALTSGVHTFPAVLQSSGL YSLSSVVTVPSSSLGTQTYICNVNHKPSNTKVDKKVEPKSCDKTHTCPPCPAPE**AA**GGPSVF LFPPKPKDTLMISRTPEVTCVVVDVSHEDPEVKFNWYVDGVEVHNAKTKPREEQYNSTYRVV SVLTVLHQDWLNGKEYKCKVSNKAL**G**APIEKTISKAKGQPREPQVYTLPPSREEMTKNQVSL TCLVKGFYPSDIAVEWESNGQPENNYKTTPPVLDSDGSFFLYSKLTVDKSRWQQGNVFSCS VMHEALHNHYTQKSLSLSPGK	Schlothauer et al., 2016 (peds)
	Kappa light chain	N/A	RTVAAPSVFIFPPSDEQLKSGTASVVCLLNNFYPREAKVQWKVDNALQSGNSQESVTEQDSK DSTYSLSSTLTLSKADYEKHKVYACEVTHQGLSSPVTKSFNRGEC	pfuse2-hclk (Invivogen)
Rabbit IgG	IgG1 heavy chain	Wild-type	GQPKAPSVFPLAPCCGDTPSSTVTLGCLVKGYLPEPVTVTWNSGTLTNGVRTFPSVRQSSG LYSLSSVVSVTSSSQPVTCNVAHPATNTKVDKTVAPSTCSKPTCPPPELLGGPSVFIFPPKPK DTLMISRTPEVTCVVVDVSEDDPEVQFTWYINNEQVRTARPPLREQQFNSTIRVVSTLPIAHE DWLRGKEFKCKVHNKALPAPIEKTISKARGQPLEPKVYTMGPPREELSSRSVSLTCMINGFYP SDISVEWEKNGKAEDNYKTTPAVLDSDGSYFLYSKLSVPTSEWQRGDVFTCSVMHEALHNH YTQKSISRSPGK	L29172 (IMGT)
	Kappa light chain	N/A	RDPVAPSVLLFPPSKEELTTGTATIVCVANKFYPSDITVTWKVDGTTQQSGIENSKTPQSPED NTYSLSSTLSLTSAQYNSHSVYTCEVVQGSASPIVQSFNRGDC	X00232 (IMGT)
Pig IgG	IgG1 heavy chain	Wild-type	APKTAPSVYPLAPCGRDTSGPNVALGCLASSYFPEPVTMTWNSGALTSGVHTFPSVLQPSG LYSLSSMVTVPASSLSSKSYTCNVNHPATTTKVDKRVGTKTKPPCPICPGCEVAGPSVFIFPP KPKDTLMISQTPEVTCVVVDVSKEHAEVQFSWYVDGVEVHTAETRPKEEQFNSTYRVVSVLP IQHQDWLKGKEFKCKVNNVDLPAPITRTISKAIGQSREPQVYTLPPPAEELSRSKVTVTCLVIG FYPPDIHVEWKSNGQPEPEGNYRTTPPQQDVDGTFFLYSKLAVDKARWDHGETFECAVMH EALHNHYTQKSISKTQGK	AB699686 (IMGT)
	Kappa light chain	N/A	RADAKPSVFIFPPSKEQLETQTVSVVCLLNSFFPREVNVKWKVDGVVQSSGILDSVTEQDSK DSTYSLSSTLSLPTSQYLSHNLYSCEVTHKTLASPLVKSFSRNECEA	FP312898 (IMGT)
