# Supplementary material for: Binding affinities of human IgG1 and chimerized pig and rabbit derivatives to human, pig and rabbit Fc gamma receptor IIIA
Source: PLoS One. 2019 Jul 19;14(7):e0219999. doi: 10.1371/journal.pone.0219999 (PMC6641210; doi:10.1371/journal.pone.0219999)
Supplement: S2 Table — (PDF) [file pone.0219999.s002.pdf]

S2 Table: FcγRIIIA sequences

**Protein**	**Extracellular Domain sequence (signal sequence underlined)**	**Sequence reference**
Human FcγRIIIA-F158 (Low affinity allele)	MWQLLLPTALLLLVSAGMRTEDLPKAVVFLEPQWYRVLEKDSVTLKCQGAYSPEDNSTQWFHNESLISSQAS<br>SYFIDAATVDDSGEYRCQTNLSTLSDPVQLEVHIGWLLLQAPRWVFKEEDPIHLRCHSWKNTALHKVTYLQNG<br>KGRKYFHHNSDFYIPKATLKDSGSYFCRGL**F**GSKNVSSETVNITITQGLAVSTISSFFPPGYQ	P08637 (Uniprot)
Human FcγRIIIA-V158 (High affinity allele)	MWQLLLPTALLLLVSAGMRTEDLPKAVVFLEPQWYRVLEKDSVTLKCQGAYSPEDNSTQWFHNESLISSQAS<br>SYFIDAATVDDSGEYRCQTNLSTLSDPVQLEVHIGWLLLQAPRWVFKEEDPIHLRCHSWKNTALHKVTYLQNG<br>KGRKYFHHNSDFYIPKATLKDSGSYFCRGL**V**GSKNVSSETVNITITQGLAVSTISSFFPPGYQ	P08637, dbSNP:rs396991 (Uniprot)
Rabbit FcγRIIIA	MGQPLPPVALLLLVSASSRAADVPKALVLLDPPWASVLKDDHVTLKCQGLHPAGDNTTQWLHNGSLLSSQAP<br>AYTITAARAEDGGEYRCQTGLSSLSDPVQLHVHLGWLVLQAPRWVFQEGEPIQLRCHSWKNNKLHKVTYLQ<br>NGRGLRYFHQNSDLHIPEATRNHSGSYFCRGLIGHHNMSSETVTITVQGPANPVISSSVLPWHQ	XP_002715293.1 (Genbank)
Pig FcγRIIIA	MWQLLSPTALLLLVSVPGTHAEDPPKSVVILDPPWDRLLEKDSVTLKCQGAYPPRDDSTEWRWNGTLISNKA<br>SSYSITDATVGNSGEYTCKTGLSAQSDPLRLEVYKGWLLLQAPRWVVQEGESIRLRCHTWKNITIQKVQYFQN<br>GMGKKFSHQNFEYHIPNATLKDGGSYFCRGIIKNYDLSSEPVKVTVQGSKSPSPILSFFLPWHQ	Q28942 (Uniprot)
