# Supplementary material for: Binding affinities of human IgG1 and chimerized pig and rabbit derivatives to human, pig and rabbit Fc gamma receptor IIIA
Source: PLoS One. 2019 Jul 19;14(7):e0219999. doi: 10.1371/journal.pone.0219999 (PMC6641210; doi:10.1371/journal.pone.0219999)
Supplement: S3 Table — (PDF) [file pone.0219999.s003.pdf]

S3 Table: Kinetic parameters derived from 1:1 Langmuir binding model. Average of 3 replicates are reported with standard deviation in square brackets [SD]. Concentrations tested are listed in parentheses below each FcγRIIIA. i.b., insufficient binding.

Ligand	Analyte (Concentrations tested in nM)	Capture Amount [SD] (RU)	k_a [SD] (1/Ms)	k_d [SD] (1/s)	K_D [SD] (M)	R_{max} [SD] (RU)	Chi^2 (RU^2)	% R_{max} of est. R_{max}
hIgG1	**hFcγRIIIA-V158** (15, 44, 132, 396, 1189)	150.0 [0.4]	1.89E+06 [1.45E+06]	0.22 [0.17]	1.20E-07 [3.08E-09]	28.77 [0.09]	1.12	76%
	**hFcγRIIIA-F158** (44, 132, 396, 1189, 3568)	162.0 [9.7]	2.66E+05 [3.31E+04]	0.16 [0.01]	6.05E-07 [2.46E-08]	35.63 [0.79]	2.72	87%
	**pFcγRIIIA** (502, 1507, 4520, 13560, 40680)	132.4 [0.3]	5.92E+03 [8.87E+02]	0.05 [0.00]	8.06E-06 [1.81E-06]	19.87 [0.85]	5.91	75%
	**rFcγRIIIA** (73, 220, 661, 1982, 5947)	169.8 [6.4]	1.22E+05 [6.94E+03]	0.33 [0.00]	2.71E-06 [1.34E-07]	30.81 [0.44]	0.77	72%
hIgG1-SD/IE	**hFcγRIIIA-V158** (1.6, 4.9, 15, 44, 132)	158.6 [0.3]	1.01E+07 [1.90E+05]	0.01 [0.00]	1.18E-09 [1.06E-11]	30.07 [0.15]	0.37	75%
	**hFcγRIIIA-F158** (1.6, 4.9, 15, 44, 132)	147.4 [8.1]	1.16E+07 [2.11E+06]	0.05 [0.00]	4.60E-09 [5.40E-10]	30.26 [0.18]	0.83	81%
	**pFcγRIIIA** (56, 167, 502, 1507, 4520)	178.7 [0.5]	4.80E+05 [1.06E+05]	0.35 [0.05]	7.26E-07 [5.30E-08]	33.40 [0.61]	1.78	94%
	**rFcγRIIIA** (8, 24, 73, 220, 661)	169.1 [0.5]	1.80E+06 [5.14E+04]	0.27 [0.01]	1.48E-07 [3.20E-09]	29.85 [0.22]	0.34	70%
hIgG1-LA/LA/PG	**hFcγRIIIA-V158** (1189, 3568, 10705)	177.4 [5.7]	i.b.	i.b.	i.b.	9.57	i.b.	21%
	**hFcγRIIIA-F158** (1189, 3568, 10705)	177.4 [5.7]	i.b.	i.b.	i.b.	11.23	i.b.	25%
	**pFcγRIIIA** (4520, 13560, 40680)	467.8 [0.4]	i.b.	i.b.	i.b.	10.46	i.b.	11%
	**rFcγRIIIA** (1189, 3568, 10705)	177.4 [5.7]	i.b.	i.b.	i.b.	6.88	i.b.	15%
pIgG1	**hFcγRIIIA-V158** (1189, 3568, 10705)	139.4 [0.5]	i.b.	i.b.	i.b.	14.84	i.b.	42%
	**hFcγRIIIA-F158** (1189, 3568, 10705)	139.4 [0.5]	i.b.	i.b.	i.b.	12.08	i.b.	34%
	**pFcγRIIIA** (167, 502, 1507, 4520, 13560)	155.8 [3.8]	5.97E+04 [3.63E+03]	0.28 [0.01]	4.73E-06 [1.96E-07]	27.01 [0.24]	1.33	87%
	**rFcγRIIIA** (1189, 3568, 10705)	139.4 [0.5]	i.b.	i.b.	i.b.	7.60	i.b.	22%
rIgG	**hFcγRIIIA-V158** (15, 44, 132, 396, 1189)	144.5 [0.3]	1.44E+06 [5.31E+05]	0.26 [0.09]	1.81E-07 [6.84E-09]	28.98 [0.20]	1.60	79%
	**hFcγRIIIA-F158** (44, 132, 396, 1189, 3568)	137.9 [0.5]	1.86E+05 [2.51E+03]	0.14 [0.01]	7.73E-07 [6.82E-08]	30.67 [0.54]	2.57	88%
	**pFcγRIIIA** (502, 1507, 4520, 13560, 40680)	137.9 [0.5]	7.11E+03 [5.06E+02]	0.08 [0.00]	1.09E-05 [1.18E-06]	28.53 [1.04]	5.36	104%
	**rFcγRIIIA** (73, 220, 661, 1982, 5947)	156.2 [0.4]	1.40E+05 [5.57E+03]	0.34 [0.02]	2.45E-06 [2.49E-08]	31.79 [0.10]	1.27	80%
