# Supplementary material for: Binding affinities of human IgG1 and chimerized pig and rabbit derivatives to human, pig and rabbit Fc gamma receptor IIIA
Source: PLoS One. 2019 Jul 19;14(7):e0219999. doi: 10.1371/journal.pone.0219999 (PMC6641210; doi:10.1371/journal.pone.0219999)
Supplement: S4 Table — (PDF) [file pone.0219999.s004.pdf]

S4 Table: Affinity values derived from steady-state binding analysis. Average of 3 replicates are reported with standard deviation in square brackets [SD]. Concentrations tested are listed in parenthesis below each FcγRIIIA.

Ligand	Analyte (Concentrations tested in nM)	Capture Amount [SD] (RU)	K_D [SD] (M)	R_{max} [SD] (RU)	Chi^2 (RU^2)	% R_{max} of est. R_{max}
hIgG1	**hFcγRIIIA-V158** (15, 44, 132, 396, 1189)	150.0 [0.4]	1.23E-07 [5.52E-10]	29.46 [0.12]	0.53	78%
	**hFcγRIIIA-F158** (44, 132, 396, 1189, 3568)	162.0 [9.7]	6.12E-07 [5.49E-08]	37.13 [1.45]	0.26	90%
	**pFcγRIIIA** (502, 1507, 4520, 13560, 40680)	132.4 [0.3]	1.47E-05 [3.14E-06]	25.11 [1.38]	1.21	95%
	**rFcγRIIIA** (73, 220, 661, 1982, 5947)	169.8 [6.4]	2.17E-06 [3.18E-08]	28.73 [0.19]	0.15	67%
hIgG1-SD/IE	**pFcγRIIIA** (56, 167, 502, 1507, 4520)	178.7 [0.5]	6.38E-07 [1.95E-08]	33.35 [0.32]	0.11	94%
	**rFcγRIIIA** (8, 24, 73, 220, 661)	169.1 [0.5]	1.33E-07 [3.93E-09]	29.26 [0.38]	0.13	68%
pIgG1	**pFcγRIIIA** (167, 502, 1507, 4520, 13560)	155.8 [3.8]	3.97E-06 [1.22E-07]	26.64 [0.20]	0.07	86%
rIgG	**hFcγRIIIA-V158** (15, 44, 132, 396, 1189)	144.5 [0.3]	1.72E-07 [4.11E-09]	29.32 [0.17]	0.39	80%
	**hFcγRIIIA-F158** (44, 132, 396, 1189, 3568)	137.9 [0.5]	7.92E-07 [4.65E-08]	32.40 [0.08]	0.08	93%
	**pFcγRIIIA** (502, 1507, 4520, 13560, 40680)	137.9 [0.5]	1.32E-05 [7.65E-07]	31.91 [0.49]	0.76	116%
	**rFcγRIIIA** (73, 220, 661, 1982, 5947)	156.2 [0.4]	1.99E-06 [4.01E-08]	30.05 [0.16]	0.04	76%
