# Supplementary material for: Binding affinities of human IgG1 and chimerized pig and rabbit derivatives to human, pig and rabbit Fc gamma receptor IIIA
Source: PLoS One. 2019 Jul 19;14(7):e0219999. doi: 10.1371/journal.pone.0219999 (PMC6641210; doi:10.1371/journal.pone.0219999)
Supplement: S1 Fig — (PDF) [file pone.0219999.s005.pdf]

S1 Fig

A

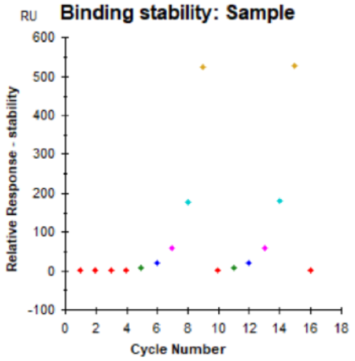

hIgG1 Concentration (nM)	Average Relative Response (RU)	% CV
0	-1.0	16%
0.2	5.4	1.4%
0.6	18.3	1.7%
1.8	58.2	0.6%
5.3	176.7	0.8%
16.2	523.4	0.2%

B

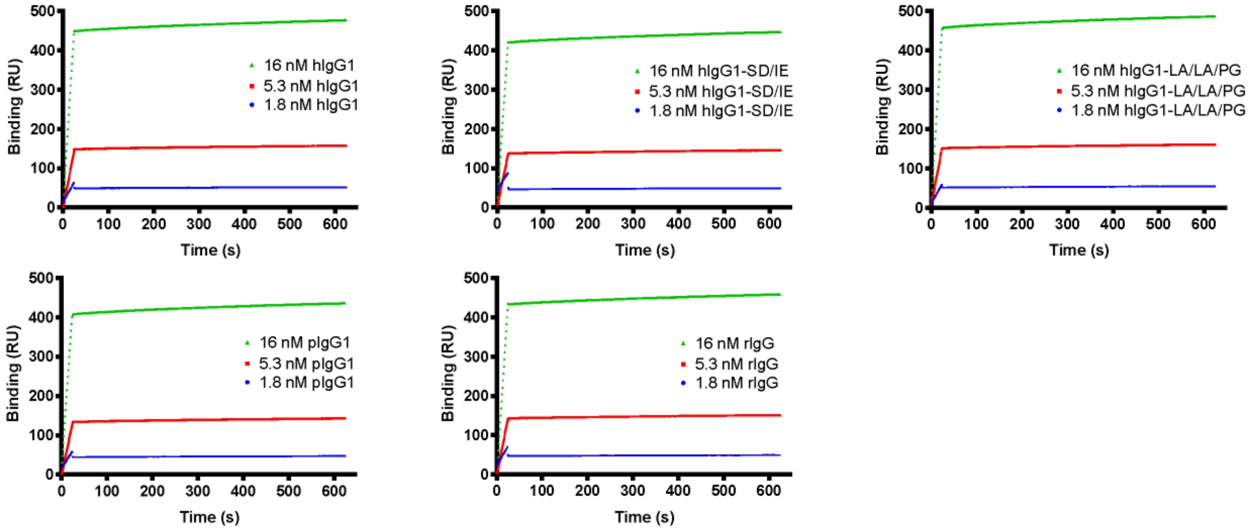

C

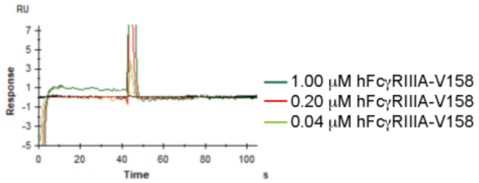

S1 Fig: Characterization of Protein L sensor chip **A,** Binding stability of capture on flow cell (FC) 2 at different concentrations of hIgG1 with regeneration between each cycle. Relative response (i.e. FC1 response subtracted from FC2 response) plotted against cycle number. The average relative response and % coefficient of variation (CV) of two replicates is reported for non-zero concentrations of hIgG1. Buffer control (hIgG1 = 0) average is determined from 6 injections. **B,** Capture of hIgG1, hIgG1-SD/IE, hIgG1-LA/LA/PG, pIgG1, and rIgG to Protein L surface by injection onto the FC2 surface for 24 s and dissociation for 600 s. **C,** Lack of nonspecific interaction between hFcγRIIIA-V158 and Protein L surface performed at a flow rate of 30 μl/min, with association for 45 s and dissociation for 60 s.
