# Supplementary material for: Binding affinities of human IgG1 and chimerized pig and rabbit derivatives to human, pig and rabbit Fc gamma receptor IIIA
Source: PLoS One. 2019 Jul 19;14(7):e0219999. doi: 10.1371/journal.pone.0219999 (PMC6641210; doi:10.1371/journal.pone.0219999)
Supplement: S2 Fig — (PDF) [file pone.0219999.s006.pdf]

S2 Fig

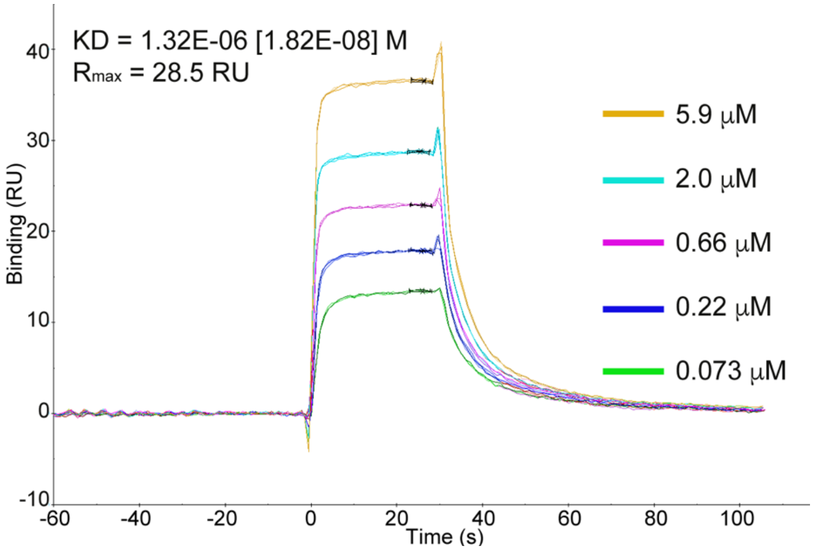

S2 Fig: Steady-state affinity of rabbit IgG to rabbit FcγRIIIA using SA sensor chip. Rabbit FcγRIIIA was injected over the surface for 30 s at concentrations listed on the right-hand side of the sensorgrams and allowed to dissociate for 60 s. Curves shown are double-referenced from buffer injections and a reference surface. Report points used for steady-state affinity analysis are indicated with black x's on curves and the average of three K_D measurements is shown in the left-top corner with standard deviation in square brackets.
