# Supplementary material for: Binding affinities of human IgG1 and chimerized pig and rabbit derivatives to human, pig and rabbit Fc gamma receptor IIIA
Source: PLoS One. 2019 Jul 19;14(7):e0219999. doi: 10.1371/journal.pone.0219999 (PMC6641210; doi:10.1371/journal.pone.0219999)
Supplement: S3 Fig — Clustal omega alignment of hIgG1, rIgG, and pIgG1 heavy chain (A) and light chain (B) constant regions with constant region numbering based on the human heavy and light chains. (PDF) [file pone.0219999.s007.pdf]

S3 Fig: Clustal Omega alignment of hIgG1, rIgG, and pIgG1 heavy chain **(A)** and light chain **(B)** constant regions with constant region numbering based on the human heavy and light chains. An asterisk (*) indicates a fully conserved residue, a colon (:) indicates strong similarity between residues, and a period (.) indicates weakly similar properties between residues. Bold and underlined, contact interface with Fc region of hIgG1 [1]. Contact interface residues conserved in rabbit and pig also bold and underlined.

A. IgG1 heavy chain Clustal Omega Alignment:

```
human      ASTKGPSVFPLAPSSKSTSGGTAALGCLVKDYFPEPVTVSWNSGALTSGVHTFPAVLQSS   177
rabbit     GQPKAPSVFPLAPCCGDTPSSTVTLGCLVKGYLPEPVTVTWNSGTLTNGVRTFPSVRQSS   177
pig        APKTAPSVYPLAPCGRDTSGPNVALGCLASSYFPEPVTMTWNSGALTSGVHTFPSVLQPS   177
           .  ..***:****.  .* . ..:****...*:*****::****:**.**:***:* * *

human      GLYSLSSVVTVPSSSLGTQTYICNVNHKPSNTKVDKKVEPKSCDKTHTCPPCPAPELLGG   237
rabbit     GLYSLSSVVSVTSS---SQPVTCNVAHPATNTKVDKTVAPSTCSK----PTCPPPELLGG   237
pig        GLYSLSSMVTVPASSLSSKSYTCNVNHPATTTKVDKRVGTKTKPP---CPICPGCE-VAG   237
           *******:*:* :*   ::   *** *  :.***** *  .:       * **  * :.*

human      PSVFLFPPKPKDTLMISRTPEVTCVVVDVSHEDPEVKFNWYVDGVEVHNAKTKPREEQYN   297
rabbit     PSVFIFPPKPKDTLMISRTPEVTCVVVDVSEDDPEVQFTWYINNEQVRTARPPLREQQFN   297
pig        PSVFIFPPKPKDTLMISQTPEVTCVVVDVSKEHAEVQFSWYVDGVEVHTAETRPKEEQFN   297
           ****:************:************.:. **:*.**::. :*:.*.   :*:*:*

human      STYRVVSVLTVLHQDWLNGKEYKCKVSNKALPAPIEKTISKAKGQPREPQVYTLPPSREE   357
rabbit     STIRVVSTLPIAHEDWLRGKEFKCKVHNKALPAPIEKTISKARGQPLEPKVYTMGPPREE   357
pig        STYRVVSVLPIQHQDWLKGKEFKCKVNNVDLPAPITRTISKAIGQSREPQVYTLPPPAEE   357
           ** ****.* : *:***.***:**** *  ***** :***** **  **:***: *  **

human      MTKNQVSLTCLVKGFYPSDIAVEWESNGQP--ENNYKTTPPVLDSDGSFFLYSKLTVDKS   415
rabbit     LSSRSVSLTCMINGFYPSDISVEWEKNGKA--EDNYKTTPAVLDSDGSYFLYSKLSVPTS   415
pig        LSRSKVTVTCLVIGFYPPDIHVEWKSNGQPEPEGNYRTTPPQQDVDGTFFLYSKLAVDKA   415
           ::  .*::**:: **** ** ***:.**:   *.**:***   * **::******:* .:

human      RWQQGNVFSCSVMHEALHNHYTQKSLSLSPGK       447
rabbit     EWQRGDVFTCSVMHEALHNHYTQKSISRSPGK       447
pig        RWDHGETFECAVMHEALHNHYTQKSISKTQGK       447
           .*::*:.* *:**************:* : **
```

B. Kappa light chain constant region:

```
human      RTVAAPSVFIFPPSDEQLKSGTASVVCLLNNFYPREAKVQWKVDNALQSGNSQESVTEQD   167
rabbit     RDPVAPSVLLFPPSKEELTTGTATIVCVANKFYPSDITVTWKVDGTTQQSGIENSKTPQS   167
pig        RADAKPSVFIFPPSKEQLETQTVSVVCLLNSFFPREVNVKWKVDGVVQSSGILDSVTEQD   167
           *  . ***::****.*:* : *.::**: *.*:* : .* ****.. *...  :* * *.

human      SKDSTYSLSSTLTLSKADYEKHKVYACEVTHQGLSSPVTKSFNRGEC--       214
rabbit     PEDNTYSLSSTLSLTSAQYNSHSVYTCEVV-QGSASPIVQSFNRGDC--       214
pig        SKDSTYSLSSTLSLPTSQYLSHNLYSCEVTHKTLASPLVKSFSRNECEA       214
            :*.********:* .::* .*.:*:***. :  :**:.:**.*.:*
```
