# Supplementary material for: Binding affinities of human IgG1 and chimerized pig and rabbit derivatives to human, pig and rabbit Fc gamma receptor IIIA
Source: PLoS One. 2019 Jul 19;14(7):e0219999. doi: 10.1371/journal.pone.0219999 (PMC6641210; doi:10.1371/journal.pone.0219999)
Supplement: S4 Fig — (PDF) [file pone.0219999.s008.pdf]

**S4 Fig:** Clustal Omega alignment of hFcγRIIIA-V158, pFcγRIIIA, and rFcγRIIIA ECD fragments. An asterisk (*) indicates a fully conserved residue, a colon (:) indicates strong similarity between residues, and a period (.) indicates weakly similar properties between residues. Bold and underlined, contact interface of hFcγRIIIA-V158 with Fc region of hIgG1 [1], using alternate numbering for hFcγRIIIA-V158 [2]. Contact interface amino acids conserved in rabbit and pig also bold and underlined. Note that Asp129 in hFcγRIIIB is Gly129 in hFcγRIIIA, rFcγRIIIA and pFcγRIIIA [2]. Bold, N-linked glycosylation sites reported in hFcγRIIIA.

```
hFcγRIIIA-V158                         RTEDLPKAVVFLEPQWYRVLEKDSVTLKCQGAYSPEDNSTQ       41
rFcγRIIIA                              RAADVPKALVLLDPPWASVLKDDHVTLKCQGLHPAGDNTTQ       41
pFcγRIIIA                              HAEDPPKSVVILDPPWDRLLEKDSVTLKCQGAYPPRDDSTE       41
                                       :: * **::*:*:* *  :*:.* ******* :   *::*:

hFcγRIIIA-V158      WFHNESLISSQASSYFIDAATVDDSGEYRCQTNLSTLSDPVQLEVHIGWLLLQAPRWVFK       101
rFcγRIIIA           WLHNGSLLSSQAPAYTITAARAEDGGEYRCQTGLSSLSDPVQLHVHLGWLVLQAPRWVFQ       101
pFcγRIIIA           WRWNGTLISNKASSYSITDATVGNSGEYTCKTGLSAQSDPLRLEVYKGWLLLQAPRWVVQ       101
                    *  * :*:*.:* :* *  * . :.*** *:*.**: ***::*.*: ***:*******.:

hFcγRIIIA-V158      EEDPIHLRCHSWKNTALHKVTYLQNGKGRKYFHHNSDFYIPKATLKDSGSYFCRGLVGSK       161
rFcγRIIIA           EGEPIQLRCHSWKNNKLHKVTYLQNGRGLRYFHQNSDLHIPEATRNHSGSYFCRGLIGHH       161
pFcγRIIIA           EGESIRLRCHTWKNITIQKVQYFQNGMGKKFSHQNFEYHIPNATLKDGGSYFCRGIIKNY       161
                    * : *:****:***  ::** *:*** * :: *:* : :**:** :..*******::

hFcγRIIIA-V158      NVSSETVNITITQGLAVSTISSFFPPGYQ     190
rFcγRIIIA           NMSSETVTITVQGPANP-VISSSVLPWHQ     190
pFcγRIIIA           DLSSEPVKVTVQGSKSPSPILSFFLPWHQ     190
                    ::*** *.:*:        * * . * :*
```

## References


1. Sondermann P, Huber R, Oosthuizen V, Jacob U. The 3.2-A crystal structure of the human IgG1 Fc fragment-Fc gammaRIII complex. *Nature.* 2000;406(6793):267-273.
2. Radaev S, Motyka S, Fridman WH, Sautes-Fridman C, Sun PD. The structure of a human type III Fcgamma receptor in complex with Fc. *J Biol Chem.* 2001;276(19):16469-16477.
